# Challenges faced by visually impaired individuals from the perspective of faculty members: a phenomenological study

**DOI:** 10.3389/fpsyg.2025.1651597

**Published:** 2025-10-15

**Authors:** Yasir Ayed Alsamiri

**Affiliations:** Department of Education, Islamic University of Madinah, Madinah, Saudi Arabia

**Keywords:** visual impairment, inclusive pedagogy, higher education, phenomenological study, accessibility policy

## Abstract

Visually impaired students in higher education face significant academic, social, and psychological barriers that are often overlooked by faculty. This phenomenological study explored these challenges at Hail University, Saudi Arabia, by purposively sampling six faculty members from the Colleges of Law and Education to capture diverse disciplinary perspectives and ranks. Semi-structured interviews were audio-recorded, transcribed, and subjected to inductive thematic analysis with member checking and inter-rater reliability to ensure rigor. Three interrelated themes emerged: Academic Challenges—including inaccessible textbooks (lack of Braille and poorly tagged digital formats), rigid visual-based teaching methods, and absence of clear accommodation policies; Social Challenges—marked by peer ignorance, campus isolation, and exclusion from collaborative learning; and Psychological Challenges—manifested in faculty feelings of helplessness and student anxiety and low self-confidence stemming from repeated accessibility failures. Contrasting with Western models that centralize disability services, Hail University’s decentralized approach places the accommodation burden on individual faculty, underscoring the need for systemic reforms. We recommend establishing dedicated disability offices, mandating the concept of education training, launching campus-wide awareness campaigns, and integrating targeted mental-health support to foster equitable and sustainable learning environments.

## 1 Introduction

The World Health Organization has adopted the International Classification of Diseases 11 (ICD 11) definition of visual impairment and blindness. According to this definition, a person is said to be visually impaired if the presenting VA in the better eye is worse than 3/60 (Kv and Vijayalakshmi, 2020). Visual impairment is a generic term used to describe a wide range of visual problems (Rahman, 2019). International Statistical Classification of Diseases and Related Health Problems (ICD) defines visual impairment categories primarily on the basis of recommendations made by a World Health Organization (WHO) Study Group in 1972 (World Health Organization, 2019) and defines as: low vision is defined as visual acuity of less than 6/18, but equal to or better than 3/60, or a corresponding visual field loss to less than 20 degrees in the better eye with best possible correction. Blindness is defined as visual acuity of less than 3/60, or a corresponding visual field loss to less than 10 degrees in the better eye with best possible correction. Aldakhil et al. (2025) reported that approximately 596 million people globally had VI, with 43.3 million of them being blind, and the majority of those affected resided in poor nations. In rural communities of Saudi Arabia, the reported prevalence of VI ranges between 13.9 and 32.1% (Aldakhil, 2025). Visually impaired students in Saudi Arabia are diagnosed through several methods, including comprehensive medical examinations, the use of visual perception scales, functional vision assessment, and observation of students’ behaviors and responses (Maliki, 2021).

One of the first disability groups to be integrated into mainstream education at the three primary levels, and also into higher education in the Kingdom of Saudi Arabia, was students with visual impairments. Historically, there have been attempts and personal efforts by interested individuals to provide educational services for students with visual impairments in the Kingdom of Saudi Arabia. However, the true beginning of the official enrollment of students with visual impairments in Saudi schools is considered to have been in the 1960s. At that time, approximately 100 blind and visually impaired students received educational services in public schools alongside students without disabilities. Also in the 1960s, the first private institute for students with visual impairments was opened, known as the Al-Noor Institute, with 40 students initially accepted. At the university level, King Saud University was the first Saudi university to allow students with visual impairments to study, in 1980, initially accepting only four students (Maliki, 2021). Since then, other Saudi universities have continued to accept students with visual impairments until the practice became widespread.

Visual impairment presents significant educational and social challenges in university settings, particularly given the heavy reliance on visual content in academic activities. As more students with disabilities enroll in higher education, ensuring equitable access has become a key issue in promoting social justice and sustainable development. According to UNESCO (2023), more than 50% of students with disabilities in developing countries still face enrollment barriers due to inadequate infrastructure (Malinovskiy et al., 2024). Therefore, it is essential to develop inclusive teaching strategies and foster supportive learning environments (Sharma, 2024). A variety of visual impairments exist within the population of students with visual impairments (Nye, 2014). Students with visual impairments are often educated in the public school setting (Kamei-Hannan et al., 2012), which requires educators to be knowledgeable of their needs. Not only do these needs include common academic curriculum, the expanded core curriculum (ECC) also needs to be purposefully taught to these learners (Lohmeier et al., 2009). There is minimal research regarding best practices and teaching strategies for these learners in the regular education classroom, resulting in a gap in practice. There is a reduced amount of research regarding regular education teachers’ perceptions of their experiences with braille and enlarged print literacy for students who are visually impaired. This study aims to offer practical recommendations to enhance the educational experiences of visually impaired students.

Saudi Arabian universities have admitted students with disabilities since the mid-1980s (Ajaj, 2022). The government has implemented several policy actions to support the development of high education opportunities for people with disabilities, including the first Legislation on Disability (LD) in 1987 and the signing of the Convention on the Rights of Persons with Disabilities (CRPD) in 2008 (Al-Eidarous et al., 2024). There is a noticeable shift toward integrating assistive technologies, especially in normative examinations (Ajaj, 2022). These exams involve assessing handwritten corrections for multiple-choice test answers extracted from scanned answer sheets. This reflects a broader movement within academia to leverage advanced technologies to make the examination process more efficient and accessible (Al-Eidarous et al., 2024).

Previous literature has agreed that there are specific, essential factors that ensure the success and progress of students with visual impairments in their academic lives. These factors are based on providing appropriate services in three main aspects: educational, technological, and infrastructure. By providing appropriate support for these basic aspects, in addition to other subsidiary aspects, it can be said that students with visual impairments are able to complete their education in a natural manner, and that many difficulties and challenges will be overcome (Al-Muqdad and Al-Qatawneh, 2018). The educational aspect is considered one of the most important aspects for all students with visual impairments, as providing educational support aims to adapt and modify educational content and offer special educational services so that they can achieve full benefit and educational progress in line with their capabilities and abilities (Al-Khalidi, 2020). This will, of course, ultimately lead to helping students with visual impairments complete their educational journey smoothly and comfortably.

Universal Design of Instruction encourages resourceful and inclusive pedagogy by presenting curriculum design and learning environments which can be fully adapted to accommodate the diversity of students with disabilities at university (Singh and Suknunan, 2023). Engaging visually impaired residents in the design process ensured tailored solutions that empower independence and well-being (Patil and Raghani, 2025). Snellen chart was used to assess Visual impairment among the students. An eye shield was needed for the younger age to cover there’s eye during the examination. The World Health Organization classifications of visual impairment were used to classify children with visual impairment: 20/30 to 20/60 is considered a near-normal vision; 20/70 to 20/160 is considered moderate visual impairment; 20/200 to 20/400 is considered severe visual impairment, and a person with 20/200 in the better eye is considered blind (Ivleva et al., 2023; Khouj et al., 2023).

Saudi Arabia has mandated inclusive higher education practices that provide equal opportunities for students with disabilities, including visual impairments (Ministry of Education, 2002). A specific “disability code” was enacted by the Saudi government in 2000 to ensure that persons with disabilities have access to free medical, educational, psychological, rehabilitation, and social services provided by public institutions (Ministry of Education, 2015). Such policy directives require that persons with disabilities have equal rights as well as free access to meaningful education as defined in the *Teacher’s Guide for Learning Disabilities* (Ministry of Education, 2015).

Public schools in Saudi Arabia have to a certain extent included disabled children in their schools. For example, the visually impaired, hearing difficulties, and less severe disabilities children were seen enrolled in public schools. Recently, the effort has increased as can be seen when the special education institutes have revised their curriculum to ensure that they are in line with the policies and practices of inclusive education in other countries. In addition, Saudi Arabia also extended the move to include opportunities to be given to children with different special educational needs and render help to those that require assistance and help. Due to these efforts, currently, Saudi Arabia is offering specialization programs, especially to children that need education and behavioral support to see them through their growing years (Al Shaban and Hanaf, 2024).

In Saudi Arabia, the Ministry of Education has implemented several initiatives to support students with disabilities. Among them is a preparatory year program, the first of its kind in the Arab world, specifically designed to help students with disabilities succeed academically. Additionally, several Saudi universities have established specialized committees—such as the Scientific Committee, the Curriculum Committee, and the Support Services Committee—dedicated to enhancing the education of individuals with disabilities (Alnajashi et al., 2023).

These committees have developed specific eligibility criteria for admitted students and have emphasized the importance of disability-focused programs that serve as academic hubs. These programs also support professionals working in the field and aim to build partnerships at the local, regional, and international levels.

Educational research shows that university students with visual impairments struggle to follow lectures without assistive tools (Marschark et al., 2015). Another research found that communication barriers often lead to social isolation and, in many cases, withdrawal from academic programs (Knoors and Marschark, 2014). Likewise, Luckner and Bowen (2006) highlighted that a lack of faculty awareness is one of the most significant challenges faced by these students. To serve its visually-impaired students better and help them achieve their educational goals, KSU has established a Blind Student Association and a Center for Special Needs Students. The Center has numerous assistive devices and software for the students to use for free such as: (a) the JAWS Screen Reader; (b) HAL Screen Reader (a voice output screen reader program); (c) Braille Sense Display and Notetakers; and (d) a Braille Embosser/Printers (Jarf, 2021). Although it has been many years since the enactment of these policy directives, they have not yet been fully implemented, especially in the context of university students with disabilities. As a consequence, there remains a lack of special needs education services for students with disabilities, including students with visual impairment (Alquraini, 2011). Barriers to implementation of special services include faculty misunderstanding or non-support of accommodations for students with disabilities (Moriña et al., 2015).

Additional studies point to the absence of updated assistive technology (Smith and Kelley, 2007), the lack of specialized academic advisors and difficulty accessing visual content such as charts or maps (Cmar et al., 2018; Kizilaslan et al., 2019). Alnahdi (2014) noted the absence of formal university policies to provide exam accommodations, while Bodaghi et al. (2017) stressed how a lack of peer awareness reinforces social exclusion.

This study focuses on the academic challenges of visually impaired students at Hail University, as perceived by faculty members. It offers the first in-depth examination of accessibility barriers in Saudi higher education. While concerns such as teaching practices and faculty training are globally relevant, this research brings unique value by investigating how institutional policy, faculty readiness, and technological infrastructure interact in the Saudi context. Understanding these challenges through a localized lens is essential for designing targeted interventions. The findings may support Saudi Arabia’s ongoing commitment to inclusive education and guide the development of standardized accessibility protocols across its higher education institutions (Alnajashi et al., 2023; Shaheen and Nazmeen, 2023).

### 1.1 Problem statement

Visual disability is a generic term used to describe a wide range of visual problems. It includes categories such as total blindness, mild and severe cases of visual impairment. The manner in which the learner uses residual vision is the main concern of educators. Based on the educational definition of visual impairment, completely blind refers to severely challenged students who must learn Braille in order to read and write, and low-vision students use their residual vision as a primary sense to deal with visual demands concerning suitable assistive devices. Lourens and Swartz (2020), Maruza et al. (2020), and Palan (2021) found that students with visual impairments face a number of challenges in higher education globally, such as access to courses’ materials, navigating the physical environment, access to technology and assistive devices, lack of awareness and understanding, social interactions, and limited opportunities. Dutta (2013) rationalized that inclusive education should facilitate access to the same information, at the same time and possibly in the same way to promote the involvement of students with visual disabilities (SWVDs) in mainstream classroom settings. To fill this gap, the current study will have in-depth interviews with faculty members of students with visual impairments in higher education at Hail University to have more insight into their opinions. It is important to investigate the faculty members’ perspectives regarding the challenges faced by their students with visual impairments in higher education at Hail University to gain an understanding of their opinions at the university.

### 1.2 Objectives

Therefore, this study aimed to investigate the challenges faced by visually impaired individuals from the perspective of faculty members.

### 1.3 Research questions

The research questions will be: What are the opinions and experiences of university academic staff regarding the educational challenges faced by students with visual impairments in higher education?

## 2 Methods

### 2.1 Research design

The research design of this study draws on phenomenology research, which involves gathering information from those who have experienced certain things, interpreting important statements in the data, and summarizing the main themes and aspects of the encounter (Plano Clark and Creswell, 2015).

### 2.2 Sample

The study sample comprised six faculty members selected via purposive sampling to ensure rich, relevant insights into teaching visually impaired students. They taught students with visual impairment. Participants were drawn from two distinct colleges (Law and Judicial Studies; Education) and spanned a range of academic ranks (Assistant to Full Professor). Purposive sampling targeted those with direct experience in inclusive pedagogy and familiarity with institutional support mechanisms, thereby maximizing data relevance. Including multiple disciplines and ranks allowed examination of how accessibility challenges differ by teaching context and level of institutional authority. A cohort of six aligns with established phenomenological guidelines recommending five to ten participants to reach thematic saturation without sacrificing depth. This size provided sufficient diversity to capture varied perspectives while permitting the sustained, iterative coding, member checking, and in-depth interviews necessary to produce nuanced, trustworthy findings suitable for rigorous academic publication. Table 1 showcases the demographic characteristics of the sample.

**Table 1:** Demographic characteristics of the sample.

Participant	College	Academic rank	Gender	Teaching experience	Committee involvement
1	College of Law and Judicial Studies	Associate Professor	Female	12 Years	Disability Services Committee
2	College of Law and Judicial Studies	Associate Professor	Male	19 Years	Curriculum Development Committee
3	College of Law and Judicial Studies	Assistant Professor	Female	5 Years	None
4	Faculty of Education	Associate Professor	Male	15 Years	Support Services Committee
5	Faculty of Education	Professor	Female	20 Years	Scientific Advisory Committee
6	Faculty of Education	Associate Professor	Male	18 Years	Inclusive Pedagogy Working Group

### 2.3 Data collection tools

Many researchers conducting qualitative studies resort to various techniques for data collection to provide a comprehensive and in-depth understanding of their research (Bahadır and Bahadır, 2024). Interviews, which are unstructured written or spoken information collected from participants in discussions when they answer open-ended questions were employed. Interviews are helpful for giving comprehensive personal information (Plano Clark and Creswell, 2015). In-depth semi-structured face-to-face interviews with the faculty members who were willing to meet, or virtually, if there was a time constraint or they were unwilling to meet face to face were conducted. Seven interviews were face-to-face, and three interviews were conducted virtually. The duration of the interviews lasted approximately 30–45 min. One-to-one interviews help get more in-depth information from the participants; virtual interviews were an option for participants who did not feel comfortable doing an interview physically, and open-ended questions gave the participants the space to explain their thoughts, feelings, and opinions.

### 2.4 Data analysis

To extract themes, the interviewees’ responses were subjected to a thematic analysis involving six stages (Dawadi, 2020): familiarizing with the data; generating initial codes; finding common themes; reviewing themes; naming and defining themes; and generating reports. Thematic analysis involves researchers actively searching for important or interesting meanings and patterns (Finlay, 2021).

### 2.5 Procedure

The interview questions were developed following a comprehensive literature review, drawing from sources such as Armstrong and Murlis (2007) and Algolayalt et al. (2023). Key focus areas included access to course materials, exam accommodations, communication with faculty, and available support resources.

Questions were open-ended to allow participants the freedom to elaborate fully. They were arranged thematically, beginning with academic materials and ending with suggestions for institutional improvement. The questionnaire was pilot-tested with visually impaired students not involved in the main study to ensure clarity, relevance, and completeness. Experts in disability studies and inclusive education also reviewed the instrument for content validity.

To ensure the trustworthiness of the data, several strategies were implemented. Member checking (or respondent validation) was conducted both during and after the interviews, allowing participants to validate the accuracy of the transcriptions and interpretations, thereby enhancing the credibility of the findings. Inter-rater reliability was established by having two researchers independently code the data, ensuring consistency and reducing potential bias in the analysis. Additionally, data triangulation was employed by aligning interview responses with existing literature and contextual observations, which contributed to a more comprehensive understanding of the research subject.

All interviews were audio-recorded, transcribed verbatim, anonymized, and analyzed using inductive content analysis. Approximately 25 open codes were identified, then grouped into 8 categories, and finally distilled into 3 central themes. The study received ethical approval from Hail University, and all participants provided informed consent.

### 2.6 Ethical considerations

This study is required to obtain approval from the Institutional Review Board (IRB) before collecting data. The author received the IRB form prior to data collection. All faculty members were asked to give their consent either through signing the consent form, which has all the information needed. Moreover, confidentiality and anonymity were assured, as the author ensured participants that their names, ages, majors, and genders would not be mentioned in the paper when they asked me not to. Furthermore, all the data from the interviews, whether it is recorded or transcribed, are securely kept in a password-protected computer and accessible only to the researcher. Additionally, all participants can withdraw from the research at any time.

### 2.7 Trustworthiness

As this study is a qualitative study, it is relevant to trustworthiness elements, which are credibility, dependability, and confirmability. Credibility is concerned with how accurate the information of the research is. To enhance the credibility of the research, the author gave the participants sufficient time to talk about their opinions; he tried not to cut them during the interview and gave them the time they needed to answer the questions. The author spent time collecting data, recording, transcribing the interviews, and using mapping techniques to create connections between the information given.

## 3 Results

Thematic analysis yielded three key themes and potential subthemes that helped inform the challenges faced by visually impaired individuals from the perspective of faculty members. Each theme is discussed below.

### 3.1 Main theme 1: academic challenges

This primary theme captures the structural and instructional barriers that inhibit visually impaired students’ full engagement in their coursework. It had following subthemes.

#### 3.1.1 Subtheme 1: inaccessible educational materials

Faculty members unanimously identified the scarcity of accessible course materials as a foundational barrier for visually impaired students. In practice, most core textbooks and readings remain available solely in print format, without Braille editions or digital files optimally formatted for screen-reading software. One associate professor explained, “*Nearly all core textbooks arrive only in print—without Braille or accessible e-formats—forcing students to rely entirely on faculty-converted notes*.” This gap compels instructors to devote substantial unpaid hours to scanning, formatting, and tagging documents manually. As another participant noted, “*We receive no financial or technical support for document conversion; I spend hours scanning and reformatting every chapter*,” highlighting both the personal toll and institutional neglect. Even when digital versions exist, they frequently lack the proper structural tagging needed by assistive technologies. A professor remarked, “*Even when materials are digitized, they are often not tagged for screen readers, so students still struggle to navigate tables and figures*.” The cumulative effect is two-fold: students confront delays in accessing essential content, and faculty experience significant workload strain. Without dedicated teams or budgets to ensure timely, high-quality conversions, course content remains effectively inaccessible, undermining both instructional equity and academic rigor.

#### 3.1.2 Subtheme 2: rigid teaching methods

Faculty members highlighted that conventional lecture formats and reliance on visual aids create significant obstacles for visually impaired students. In many courses, instructors depend heavily on PowerPoint slides, whiteboard diagrams, and in-class demonstrations without sufficient verbal narration or alternative descriptions. As one associate professor explained, “*My lectures rely heavily on PowerPoint slides—I rarely have time to verbalize every graphic element*,” illustrating how time constraints and course design limit inclusive delivery. Another participant added, “*When I draw diagrams on the board, blind students simply cannot follow; there’s no real-time description*,” underscoring the absence of systematic adaptation during live instruction. Laboratory sessions present further challenges: “*In lab sessions, I struggle to provide tactile or audio alternatives for experiments designed around visual observation*,” remarked a faculty member, emphasizing the difficulty of converting hands-on, visually driven activities into accessible formats. Because pedagogical practices are deeply entrenched in visual communication, instructors often feel unprepared and constrained, leading to inconsistent accommodations. Without institutional guidance or resources to redesign teaching methods—such as integrating descriptive narration, tactile models, or collaborative note-taking systems—visually impaired students remain unable to engage fully with core instructional content.

#### 3.1.3 Subtheme 3: absence of clear policies

Participants consistently emphasized the lack of clear, actionable institutional policies as a major barrier to supporting visually impaired students. While most universities maintain general statements about equality or inclusive education, these are often vague and lack practical implementation strategies. One professor stated, “*Our university has a broad ‘equal opportunity’ statement, but no procedures for budgeting assistive technologies*,” pointing to a disconnect between institutional rhetoric and operational support. Faculty often bear the burden of accommodation without official guidance, funding, or training. As one participant recounted, “*In Arts, one colleague funds e-books personally—there’s no central policy or funding line*,” reflecting a system reliant on individual initiative rather than institutional responsibility. This lack of structure results in uneven practices across departments, with some students receiving *ad hoc* support while others are left without essential accommodations. Another faculty member explained, “*Without written protocols, every accommodation becomes a case-by-case negotiation, creating inconsistency*.” Participants expressed concern that the absence of standardized procedures—particularly for accessible exams, assistive technology procurement, and faculty development—undermines both student success and academic equity. The findings point to an urgent need for universities to move beyond symbolic inclusion and develop enforceable policies that translate commitments into consistent, actionable practices.

### 3.2 Main theme 2: social challenges

This theme highlights the interpersonal and cultural obstacles that contribute to visually impaired students’ isolation on campus.

#### 3.2.1 Subtheme 1: campus isolation

Campus isolation refers to the physical and participatory separation experienced by visually impaired students within lecture halls, laboratories, and informal learning environments. Faculty members reported that, lacking confidence in navigating social and spatial cues, these students often choose or are relegated to seating arrangements that limit interaction. One associate professor recounted, “*I’ve seen blind students sit at the back and never speak up—they fear missing nonverbal cues*,” illustrating how seating position can become both a symptom and a cause of disengagement. Over time, this physical isolation translates into academic disadvantage: students miss spontaneous clarifications, side conversations about assignments, and informal peer tutoring that most sighted students rely upon.

Beyond the lecture hall, isolation extends to group work and study sessions. Faculty observed that sighted peers, uncertain how to accommodate a visually impaired student, may omit them from project teams or assign them marginal tasks. As one assistant professor described, “*One student was outright told by her group, ‘We do not know how to include you,’ so she stopped attending study sessions*.” This rejection has profound implications: not only does it reduce opportunities for collaborative learning, it also diminishes students’ sense of belonging and academic self-efficacy. Faculty noted that when visually impaired students are excluded from group contexts, they lose access to critical discourse, peer feedback, and co-constructions of knowledge that underpin higher-order learning.

The cumulative effect of such isolation is a cycle of withdrawal and decreased academic engagement. Faculty members expressed concern that if left unaddressed, this pattern leads to absenteeism, reduced participation in class, and, ultimately, higher dropout rates. One professor emphasized, “*Isolation does not just affect grades—it affects motivation, mental health, and the student’s entire university experience*.” Addressing campus isolation, therefore, requires proactive measures: intentional seating arrangements, structured group assignments that mandate inclusive roles, and faculty-led ice-breaker activities designed to foster early connections between visually impaired students and their peers.

#### 3.2.2 Subtheme 2: lack of awareness among peers

A prevailing barrier to social integration is the general lack of awareness and understanding among sighted students regarding visual impairment. Faculty reported that absent formal training or orientation programs, many students simply do not know how to interact respectfully and effectively with visually impaired classmates. A faculty member noted, “*No orientation or workshops teach students how to interact with blind classmates—so many simply avoid eye contact or conversation*,” highlighting a campus culture that defaults to avoidance rather than accommodation. This ignorance not only deprives visually impaired students of peer support but also perpetuates misconceptions that disability equates with inability.

Such misconceptions manifest in everyday academic interactions. For instance, group discussions often proceed at a visual pace, with participants pointing to slides or whiteboards without providing verbal descriptions. As one associate professor observed, “*Group projects become stressful for blind students; peers do not know how to explain visual content verbally*,” underscoring how small lapses in communication can compound into significant barriers. Similarly, social gatherings and extracurricular events frequently overlook accessibility considerations—flyers lack tactile or large-print formats, and event venues are selected without routing or signage for those with low or no vision. These omissions send an implicit message: visually impaired students are an afterthought in campus life.

Faculty expressed that this pervasive lack of awareness cultivates feelings of alienation among visually impaired students. An assistant professor explained, “*I’ve had to mediate conflicts where sighted students complained about ‘slowed-down’ group work because they did not understand accommodation needs*,” demonstrating how peer resentment can arise from misinformed expectations. To counteract these dynamics, interviewees recommended mandatory disability-sensitivity training for all students—integrated into orientation week and refreshed annually—to foster empathy, communication skills, and practical strategies for inclusive collaboration.

#### 3.2.3 Subtheme 3: inadequate institutional support for social integration

While peer attitudes play a central role, faculty also identified the absence of formal university mechanisms designed to promote social integration of visually impaired students. Unlike many Western institutions that employ “buddy systems,” peer-mentor programs, or disability services offices with dedicated social coordinators, Hail University lacks clear structures to guide involvement. One professor explained, “*We have no designated social liaison for blind students—everything is up to individual faculty effort*,” illustrating how the onus falls on instructors rather than a coordinated support network.

This institutional gap means that initiatives such as accessible campus tours, social skill workshops, or inclusive clubs rarely materialize. As one associate professor shared, “*I once tried to organize a campus orientation with tactile maps and audio guides, but without official backing, it never took off*.” Consequently, visually impaired students miss crucial opportunities to build friendships, develop informal support networks, and participate fully in the university’s co-curricular life. Faculty noted that these experiences are not peripheral; they contribute significantly to professional development, leadership skills, and a sense of community that enhances academic success. To remedy this, participants recommended establishing a centralized Office of Disability Inclusion with a mandate to design and implement social integration strategies. Suggested measures included a peer-mentoring program pairing sighted and blind students, regular social events with built-in accessibility features, and a campus ambassador initiative to train student leaders in inclusive practices. According to a faculty member, “*Formalizing these supports would signal institutional commitment and reduce faculty burnout from carrying the entire burden*.” Such systemic changes, faculty agreed, are essential to transform social inclusion from a sporadic, *ad hoc* endeavor into a sustainable, rights-based component of the university experience.

### 3.3 Main theme 3: psychological challenges

The third theme encompasses the emotional impacts of both academic and social barriers on faculty and students alike. It has following subthemes.

#### 3.3.1 Subtheme 1: faculty’s feelings of helplessness

Faculty members frequently described a pervasive sense of helplessness when tasked with accommodating visually impaired students, a feeling rooted in their lack of training, resources, and institutional direction. One associate professor confessed, “*I feel unqualified to teach inclusively—I’ve never had formal training on assistive software*,” capturing the uncertainty that arises when faculty must navigate unfamiliar technologies without guidance. This sentiment was echoed by a colleague who remarked, “*Allocating extra time makes me anxious about fairness; I worry other students will resent me*,” illustrating how concerns about equity can compound stress. The lack of clear procedural policies exacerbates these emotions: as one faculty member noted, “*I’m left to negotiate each accommodation case by case, which makes me feel like I’m always moving the goalposts*.” Over time, these individual struggles accumulate into professional burnout, with several instructors reporting chronic guilt over perceived shortcomings. “*Sometimes I feel guilty for not doing more, but I simply do not have the resources or time*,” admitted one professor, highlighting how inadequate support systems leave well-intentioned instructors feeling personally at fault for systemic failures. This emotional burden affects not only teaching effectiveness but also faculty morale, as instructors question their own competence and commitment. Because there is no dedicated office or specialist to whom they can refer students, professors feel isolated in their efforts. An assistant professor explained, “*I’m supposed to be an expert in my field, yet I’m fumbling through basic accessibility issues—which undermines my confidence as an educator*.” Such feelings of helplessness not only strain the faculty–student relationship but risk discouraging instructors from engaging deeply with inclusive pedagogy. Without targeted professional development, clear guidelines, and institutional investment in assistive technologies, this subtheme of helplessness will continue to impede both faculty well-being and student outcomes.

#### 3.3.2 Subtheme 1: student’s decreased self-confidence

Visually impaired students’ self-perceptions emerged as a critical psychological barrier, with faculty observing marked declines in confidence that impede academic participation and social engagement. A professor remarked, “*I see bright students shrink in confidence when they cannot keep pace with visual coursework*,” underscoring how repeated accessibility failures can lead students to internalize deficiencies that are, in fact, systemic. This erosion of self-esteem often manifests in classroom behaviors: as one participant shared, “*Many express anxiety about group work or speaking up; they internalize peer ignorance as personal failure*.” Even when accommodations are provided, students may doubt their own abilities: “*After constantly relying on audio descriptions, some students question if they truly understand course concepts*,” explained another instructor. Such self-doubt can spiral into reluctance to seek help or challenge exam results, ultimately affecting academic performance. Faculty also noted that low confidence spills into social domains, with one associate professor observing, “*Students hesitate to join study groups or campus clubs because they fear being a burden or ‘slowing down’ others*.” Anxiety about fitting in can lead to withdrawal, reinforcing social isolation and limiting opportunities for peer-supported learning. Moreover, the psychological toll extends beyond the classroom: “*I’ve had students confide that they worry they are not ‘university material*,’” a faculty member recounted, indicating how persistent exposure to inaccessible environments can damage long-term academic identity. Addressing this subtheme requires more than material accommodations; it demands dedicated confidence-building measures, such as peer mentoring, accessible study workshops, and faculty-led affirmations of student competence. Without these interventions, students’ low self-confidence perpetuates a cycle of underperformance and disengagement that undermines the broader goals of inclusion.

#### 3.3.3 Subtheme 1: inadequate psychological support services

Beyond individual feelings of helplessness and diminished self-esteem, both faculty and students identified the absence of formal psychological support services as a significant challenge. Although many universities in other regions maintain dedicated counseling units or disability resource centers, Hail University lacks an integrated support infrastructure tailored to the unique mental health needs of visually impaired students. One professor remarked, “*We have no counseling team versed in the emotional impact of disability, so students’ stress often goes unaddressed*,” illustrating the gap between academic accommodations and emotional care. Faculty members expressed concern that without professional intervention, students’ anxiety and depression symptoms remain hidden until they culminate in academic withdrawal. As one associate professor explained, “*I once referred a student to the general counseling office, but they had no experience with vision impairment and could not offer practical coping strategies*.” The result is an environment where students must navigate both academic and emotional hurdles without specialized guidance. This void also affects faculty: instructors who attempt to provide informal emotional support describe feeling ill-equipped to manage psychological crises. “*When a student breaks down in my office, I want to help, but I do not know enough about disability-specific counseling*,” admitted a participant, highlighting the risk of secondary trauma and professional stress. Moreover, the university’s omission of structured support reinforces negative self-perceptions among students; without visible commitment to mental health, they may feel their struggles are not valued. Participants recommended establishing a specialized counseling unit within the disability services framework, staffed by professionals trained in adaptive strategies for visual impairment. Proposed services include individual therapy focusing on self-advocacy, group workshops on resilience, and crisis hotlines staffed by knowledgeable counselors. By integrating psychological support with academic accommodations, the institution can foster holistic well-being, reduce attrition, and empower both students and faculty to engage more confidently in inclusive education.

## 4 Discussion

This phenomenological study examined faculty perceptions of challenges that visually impaired students encounter at a Saudi Arabian university. Through in-depth, semi-structured interviews with six purposively sampled faculty members representing diverse colleges and ranks, three primary themes emerged: (1) Academic Challenges, encompassing inaccessible materials, rigid teaching methods, and absence of clear policies; (2) Social Challenges, including campus isolation and peer unawareness; and (3) Psychological Challenges, reflected in faculty feelings of helplessness and students’ low self-confidence.

Firat’s (2021) findings in his study in Turkey stated that students with visual impairments still face many difficulties, such as unwillingness of the faculty to support students, and lack accessible materials. Alsamiri and Alduaylij (2025) found several significant barriers including inconsistent accessibility of digital course materials, with 80% of students reporting difficulties with PDF documents and presentation slideslimitations in exam accommodations, particularly insufficient time allowances and inadequate screen reader compatibility; and varying levels of faculty awareness, with only 20% of instructors proactively addressing accessibility needs. Ndonyo (2007) in a study of teacher perceptions of inclusive education found many challenges including lack of materials for teaching and learning, unfriendly infrastructure as well as learners teasing attitudes of their peers with disabilities among other challenges. The findings on first theme align closely with earlier research on higher education inclusion. For instance, the scarcity of Braille and screen-reader–compatible texts echoes Smith and Kelley’s (2007) observation that outdated or untagged materials impede learning. Similarly, faculty reports of insufficient audio descriptions and tactile adaptations in laboratories corroborate conclusion that instructional practices remain overwhelmingly visual (Burgstahler, 2020; Kizilaslan et al., 2019; Smith and Kelley, 2007; Supalo et al., 2016). At the policy level, participants’ frustration over *ad hoc* accommodations mirrors Alnahdi (2014) critique of generic equality statements lacking actionable procedures. Thus, to meet the needs of students with visual impairment, assistive technologies such as voice-activated calculators, computers with synthetic and customisable fonts, and Braille are required. Giving visually impaired students access to assistive technologies could help the inclusive education movement (Marsack, 2013).

Socially, the peer exclusion identified (as second main theme) in present research reflects Bodaghi et al. (2017) findings of cultural unawareness in academic libraries, while the campus isolation phenomenon resonates with Cmar et al. (2018), who linked group-work exclusion to higher attrition among students with visual impairments. Kasiram and Subrayen (2013) reported social exclusion of students with visual impairments. Social exclusion involved students with visual impairments being excluded from group works, hurtful comments against students with visual impairments, residences of students with disabilities labeled as nursing homes. Other challenges included absence of reasonable accommodation such as lack of recorders and lifts to high buildings. Students without disabilities were reported to be selfish, abusive and disrespectful to those with visual impairments. Social skills’ instruction was an area of reported need, as were skills in planning and use of unstructured free time for recreation and leisure. Students with disabilities in fourth year at college did not have opportunities for socialization (Corbin and Strauss, 2008). Another research done in Bangladesh claimed that disable students report difficulties in receiving alternative education, being involved in class, and facing inequality in the educational environment, compounded by a lack of educational assistive devices and unfair treatment during examinations (Hossen et al., 2023). In the United Kingdom, ableism embedded in the cultural landscape of higher education leads to negative societal constructions about visual impairment and blindness, contributing to the social challenges faced by these students (Croft, 2020).

Psychologically, the dual burden on faculty and students extends the work of Knoors and Marschark (2014), highlighting how gaps in training fuel both instructor guilt and student anxiety. Feelings of helplessness, anxiety, and diminished self-confidence often stem from repeated accessibility failures and inadequate support. Although research into the psychological impact of inaccessible environments remains limited, Kizilaslan et al. (2019) document how faculty unpreparedness aggravates student distress, and Reed and Curtis (2012) pinpoint institutional neglect as a compounding factor. Ferrando et al. (2013) and Scaffa and Reitz (2013) further demonstrate that visually impaired students experience higher rates of anxiety and depression, reinforcing cycles of self-doubt. Collectively, these studies—alongside our own—make clear that only systemic reforms, including mandated inclusive pedagogy, tactile and audio adaptations, and robust mental health services, can disrupt these destructive patterns and promote true educational equity (Wiener et al., 2010).

The primary contribution of this research lies in its divergence from many international contexts. Unlike Western institutions, which often use formal peer-mentoring programs and centralized disability service offices, Hail University’s decentralized approach places the burden of accommodation squarely on individual faculty members. This highlights a unique regional challenge: the necessity of embedding inclusive practices within institutional structures rather than depending on personal initiative. Moreover, whereas much of the global literature relies on student self-reports, our faculty-centered perspective exposes the emotional toll on instructors—an underexplored but critical aspect of sustainable inclusion.

### 4.1 Strengths and limitations

This phenomenological study offers several notable strengths alongside inherent limitations. On the strengths side, the rigorous use of member checking and inter-rater reliability procedures enhanced the credibility and trustworthiness of the thematic analysis. Purposive sampling across academic ranks and colleges provided diverse institutional perspectives, while the phenomenological design enabled an in-depth exploration of faculty lived experiences rather than superficial descriptions. However, these contributions must be weighed against certain constraints. The modest sample size of six, although in keeping with phenomenological norms, restricts the generalizability of findings beyond Hail University. Additionally, recruiting participants solely from the Colleges of Law and Education may have overlooked discipline-specific challenges present in STEM or humanities faculties. Finally, the absence of direct student interviews means that our insights into learner experiences are mediated through faculty perceptions, introducing potential observer bias. That is, there were no in-depth interviews with the visually impaired students. Therefore, the findings are mainly and strictly from faculty members’ perspectives, as the aim of this research is to explore their perspectives. Balancing these strengths and limitations provides a clear framework for interpreting the study’s contributions and guiding future research.

### 4.2 Implications

The intertwined nature of academic, social, and psychological challenges suggests that piecemeal interventions will fall short. Universities should develop and fund clear accessibility policies, establish dedicated disability-inclusion offices, and mandate inclusive-pedagogy training. Simultaneously, embedding disability awareness into student orientation and creating formal peer-mentoring programs can mitigate social isolation. Psychological support must be integrated with academic services, with counselors trained in disability-specific issues.

### 4.3 Suggestions for future research

Future research should adopt mixed-methods or longitudinal designs to assess the impact of comprehensive inclusion frameworks on student retention, academic performance, and well-being. Incorporating direct student interviews or surveys would capture lived experiences and validate faculty perceptions. Comparative studies across Saudi institutions, and between Saudi and international universities, could identify best practices adaptable to different cultural and structural contexts. By addressing these multifaceted challenges in a coordinated manner, higher-education institutions can move from rhetorical commitments to demonstrable equity, ensuring that visually impaired students thrive academically, socially, and psychologically.

## 5 Conclusion

This study examined the challenges faced by visually impaired students at Hail University through faculty perspectives, revealing three key themes: academic challenges, social challenges, and psychological challenges. Unlike Western institutions with centralized disability services, Hail University’s decentralized approach places the accommodation burden on individual faculty. To achieve true inclusion, universities must adopt systemic reforms: establish dedicated disability offices, mandate inclusive-pedagogy training, launch campus-wide awareness campaigns, and provide targeted mental-health support. Though specific to one institution, these insights offer a blueprint for higher-education environments worldwide to move from *ad hoc* measures to sustainable, equitable practices.

## Data Availability

The original contributions presented in the study are included in the article/supplementary material, further inquiries can be directed to the corresponding author.
